# The Role of Atherectomy in Lesion Preparation for Peripheral Artery Disease: Evolution of Technologies, Available Devices, and Contemporary Evidence

**DOI:** 10.31083/RCM51145

**Published:** 2026-07-24

**Authors:** Solon Antoniades, Grigorios Korosoglou

**Affiliations:** ^1^Department of Vascular and Endovascular Surgery, Isar Klinikum München, 80331 Munich, Germany; ^2^Department of Cardiology and Vascular Medicine, GRN Hospital Weinheim, 69469 Weinheim, Germany; ^3^Department of Cardiology and Vascular Medicine, GRN Hospital Eberbach, 69412 Eberbach, Germany

**Keywords:** atherectom, peripheralarterydisease, chroniclimb-threateningischemia, lesionpreparation, claudication

## Abstract

Atherectomy has become an established tool for lesion preparation in the endovascular treatment of peripheral artery disease (PAD), particularly for complex and heavily calcified lesions. Despite the widespread clinical use of atherectomy, the long-term benefits of this tool remain controversial, and the available evidence is heterogeneous. This review aimed to provide a clinically oriented, expert-driven narrative evaluation of peripheral atherectomy, including the historical evolution, currently available technologies, and the contemporary clinical role of the procedure, while integrating randomized and real-world evidence to support rational patient and lesion selection. A narrative literature review was conducted based on continuous expert monitoring since 2021 and targeted PubMed searches using combinations of the terms “peripheral artery disease”, “atherectomy”, “critical limb ischemia”, and “intermittent claudication”. Randomized controlled trials, meta-analyses, major registries, and high-quality observational studies were identified, selected based on clinical relevance and methodological quality, and synthesized accordingly. Randomized trials have consistently shown that atherectomy does not provide superior long-term patency or limb salvage compared with balloon angioplasty, stenting, or drug-coated balloons (DCB). However, atherectomy has demonstrated reproducible procedural advantages across multiple studies, including greater luminal gain, fewer dissections, and less need for bailout stenting. Observational and registry data further suggest potential benefits in selected scenarios, particularly for heavily calcified lesions, long-segment occlusions, in-stent restenosis (ISR), and anatomically challenging mobile segments. Current evidence does not support the routine use of atherectomy in PAD. Nevertheless, when applied selectively in appropriately chosen patients and lesions, atherectomy represents a valuable strategy for vessel preparation. The responsible use of atherectomy requires careful integration of the available evidence with operator expertise, awareness of alternative technologies, and consideration of procedural risks and reimbursement issues.

## 1. Introduction

Peripheral artery disease (PAD) affects a growing number of patients worldwide and is frequently associated with advanced age, multimorbidity, and impaired functional status [1]. Over the past two decades, endovascular revascularization has become the preferred treatment strategy in many clinical scenarios [2], owing to its lower perioperative morbidity and broad applicability in high-risk populations. Consequently, effective lesion preparation has emerged as a central component of contemporary endovascular therapy.

Atherectomy was developed to facilitate vessel preparation by mechanically modifying atherosclerotic plaque, improving vessel compliance, and reducing barotrauma during subsequent balloon angioplasty. Multiple atherectomy technologies—including directional, rotational, orbital, and laser-based systems—are currently available, reflecting diverse technical concepts and clinical applications. Despite widespread adoption in daily practice, the long-term clinical benefit of atherectomy remains controversial.

Randomized controlled trials have consistently demonstrated procedural advantages, such as improved acute luminal gain and reduced bailout stenting, but have failed to show clear superiority over balloon angioplasty, drug-coated balloons (DCB), or contemporary stent-based strategies with regard to long-term patency and limb-related outcomes. In contrast, real-world registries and observational studies frequently report favorable outcomes in complex lesion subsets, including heavily calcified lesions, long-segment occlusions, in-stent restenosis (ISR), and anatomically challenging segments. This apparent discrepancy between controlled trial data and daily clinical practice complicates evidence-based decision-making.

In this context, clinicians—particularly less experienced operators—are often confronted with uncertainty regarding the appropriate role of atherectomy within an increasingly complex therapeutic landscape that also includes intravascular lithotripsy (IVL), drug-coated balloons, and drug-eluting stents (DES). A balanced interpretation of available evidence, integrated with practical experience, is therefore required to guide rational patient and lesion selection.

Accordingly, this manuscript was conceived as an expert-driven, clinically oriented narrative review aiming to outline the evolution of atherectomy technologies, summarize currently available devices, synthesize randomized and real-world evidence, critically appraise benefits and limitations, and translate current knowledge into practical clinical guidance. By integrating published data with long-term clinical experience, this review seeks to support informed decision-making and responsible use of atherectomy in contemporary PAD management.

## 2. Methods

This manuscript was conceived as an expert-driven narrative review. Since mid-2021, the authors have been continuously engaged in clinical and scientific work on peripheral atherectomy and vessel preparation, during which relevant randomized trials, meta-analyses, registries, and major observational studies were systematically collected and reviewed.

In addition, targeted electronic literature searches were performed in PubMed to identify studies published between January 2000 and March 2025. Search terms included combinations of “peripheral artery disease”, “atherectomy”, “directional atherectomy”, “rotational atherectomy”, “orbital atherectomy”, “laser atherectomy”, “drug-coated balloon”, “drug-eluting stent”, and “intravascular lithotripsy”.

Reference lists of relevant reviews, meta-analyses, and international guidelines were screened to identify additional eligible studies. Publications were selected based on clinical relevance, methodological quality, and applicability to contemporary endovascular practice, with particular emphasis on randomized controlled trials and large prospective registries.

The available evidence was synthesized narratively and interpreted in the context of the authors’ clinical experience, with the aim of providing practical guidance for clinicians involved in the endovascular treatment of peripheral artery disease.

### Evolution of Atherectomy and Historical Data

The first directional atherectomy catheter was introduced by Simpson and colleagues in 1984 [3]. The device featured a cylindrical housing with a side window and an inflatable balloon that pressed the catheter against the arterial wall, allowing a motor-driven cutter to excise and collect atherosclerotic plaque. In the initial clinical experience involving 45 patients with peripheral artery disease, primary technical success (defined as residual stenosis <30%) was achieved in 88% of cases and increased to 100% when combined with adjunctive balloon angioplasty, with no major complications reported. These early results demonstrated the feasibility of controlled plaque debulking as an alternative to conventional balloon angioplasty [4].

The first rotational atherectomy device, the Kensey catheter, was introduced in 1988. It employed a high-speed rotating cam tip, operating at up to 100,000 revolutions per minute, to mechanically disrupt plaque while largely preserving the arterial wall. In an initial series of patients with femoropopliteal disease, technical success was achieved in 14 of 23 lesions, frequently in combination with balloon angioplasty and without major embolic events or periprocedural mortality [5].

Around the same period, the Rotablator system was developed and first reported in 1987. This device utilizes a diamond-coated burr rotating at high speed to selectively ablate calcified plaque while sparing more elastic arterial tissue. Experimental studies in human cadaver femoropopliteal and tibial arteries demonstrated high success rates in stenotic lesions (95%) but more limited efficacy in chronic occlusions (56%). Particle analysis revealed the generation of microparticles smaller than erythrocytes, without evidence of ischemic effects in animal models [6].

Laser-based plaque modification also emerged in the late 1980s. In 1987, percutaneous laser angioplasty using an argon laser–heated probe was first reported in femoropopliteal disease. In a small series of 13 patients (15 vessels), angiographic success was achieved in 93% and clinical success in 80% of cases, with no major complications. These early data supported the feasibility of laser-assisted recanalization as an adjunct to balloon angioplasty [7].

## 3. Atherectomy Types

Although device design has evolved substantially over the past decades, the fundamental principles of atherectomy have remained largely unchanged. Directional systems remove plaque through side-cutting windows, whereas front-cutting devices either extract material by aspiration or Archimedean screw mechanisms or pulverize it into microparticles that do not require retrieval. Laser atherectomy has evolved from early thermal recanalization techniques into modern photoablative systems that achieve tissue ablation without significant heat generation. More recently, orbital atherectomy has been introduced, utilizing a diamond-coated eccentric crown to create an elliptical orbit at high rotational speeds, enabling controlled and progressive lumen enlargement while minimizing vessel trauma [8]. In the following sections, we summarize the main characteristics and clinical evidence of the currently available atherectomy modalities.

### 3.1 Directional Atherectomy

Directional atherectomy enables selective plaque excision using a rotating cutting blade housed within a side-opening window, with excised material captured in a distal chamber. The catheter can be steered toward eccentric lesions, allowing controlled debulking while largely preserving healthy vessel wall structures.

The most widely used contemporary system is the HawkOne™ device (Medtronic, Dublin, Ireland), which represents a direct evolution of the original Simpson catheter. It features improved cutting efficiency, a lower crossing profile, and a four-blade design optimized for a broad spectrum of plaque morphologies, including severe calcification [9].

Large prospective studies have confirmed its procedural safety and efficacy. In the DEFINITIVE LE study, enrolling 800 patients with infrainguinal disease, directional atherectomy achieved a 12-month primary patency of 78% in claudicants and 95% freedom from major amputation in patients with critical limb ischemia, with a bailout stent rate of only 3.2% [10]. The DEFINITIVE Ca++ trial, focusing on moderately to severely calcified femoropopliteal lesions, reported 93% freedom from major adverse events at 30 days and a mean residual stenosis of approximately 24% after adjunctive therapy [11]. These findings highlight the ability of directional atherectomy to achieve substantial lumen gain while minimizing the need for stent implantation, even in heavily calcified disease.

The Pantheris™ OCT-guided directional atherectomy system (Avinger, Redwood City, CA, USA) integrates real-time optical coherence tomography imaging into the catheter tip, allowing direct visualization of plaque morphology and vessel wall layers. In the multicenter VISION trial, treatment of femoropopliteal lesions achieved a technical success rate of 97%, with a reduction in mean stenosis from 79% to 30% after atherectomy alone and to 22% after adjunctive therapy. The six-month target lesion revascularization rate was 6.4%, and no perforations were reported, while histological analysis confirmed minimal adventitial involvement in most samples [12]. Despite these favorable results, clinical adoption of this technology has remained limited.

**Fig. 1.**
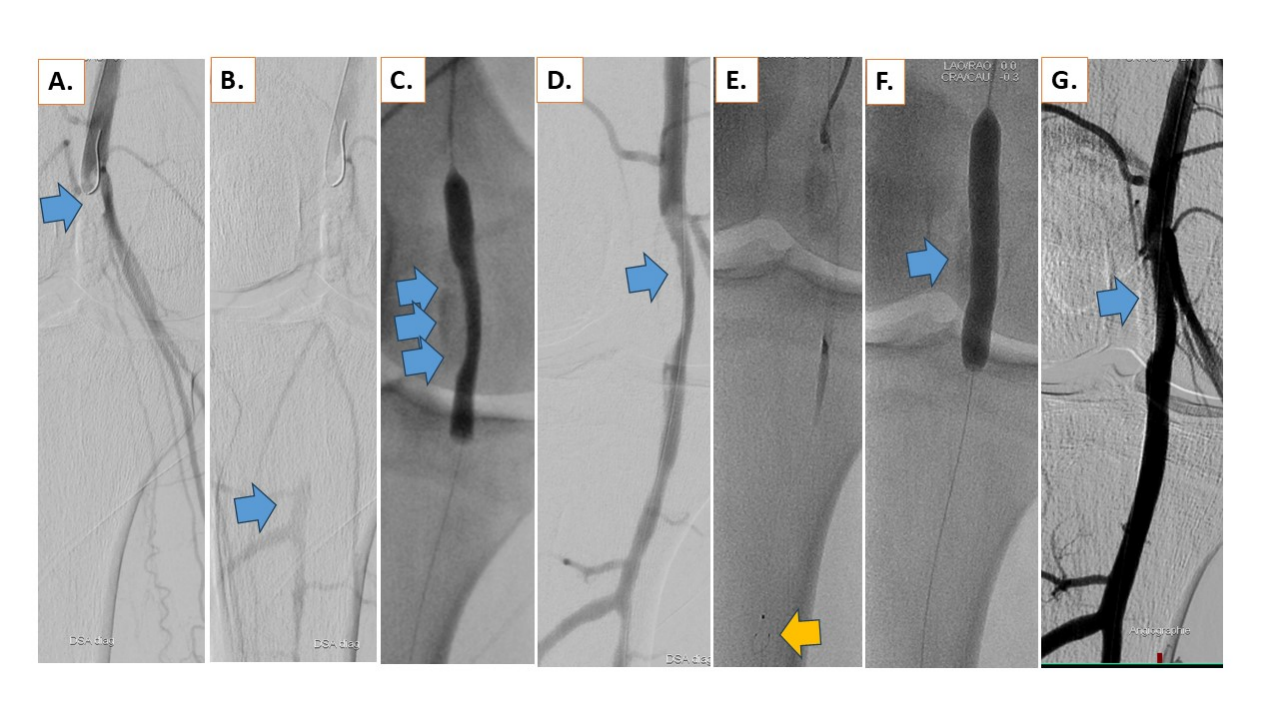
**Directional atherectomy of the popliteal artery after failed balloon angioplasty**. Directional atherectomy in a 44-year-old patient (Rutherford category 4) with popliteal artery occlusion (blue arrows in A,B depicting popliteal artery occlusion and contrast refilling in the P3 segment of the artery). After antegrade guidewire crossing, initial balloon angioplasty shows poor expansion and vessel recoil due to heavy calcification (blue arrows in C,D). Directional atherectomy using the HawkOne™ device with distal embolic protection (yellow arrow in E) enables adequate balloon expansion (blue arrow in F) and a satisfactory final result without stent placement (blue arrow in G).

An example of directional atherectomy is illustrated in Fig. 1.

### 3.2 Rotational Atherectomy

Rotational atherectomy is characterized by a front-cutting mechanism in which plaque is mechanically disrupted at the catheter tip. Device platforms differ primarily in their handling of excised material. Systems such as Jetstream (Boston Scientific, Marlborough, MA, USA) and Rotarex (Becton, Dickinson and Company, Franklin Lakes, NJ, USA) employ active aspiration, whereas the Phoenix catheter (Philips, Amsterdam, The Netherlands) utilizes an Archimedean screw for continuous debris removal. The ByCross device (Taryag Medical, Or Akiva, Northern District, Israel) integrates high-capacity aspiration and, in selected cases, enables crossing of occlusions without prior guidewire passage. In contrast, the Rotablator (Boston Scientific, Marlborough, MA, USA) pulverizes plaque into microparticles that are cleared by blood flow, although its use in peripheral arteries remains off-label. Additional systems such as Revolution (Rex Medical, L.P., Conshohocken, PA, USA) and TemRen (Invamed Vascular, Ankara, Turkey) represent further developments in this category, though with more limited published data. Of note, the Phoenix platform also offers variants with deflectable tips, enabling a partially directional mode of action.

Several studies have demonstrated the effectiveness of rotational atherectomy across different platforms. In an intravascular ultrasound–based study, Jetstream atherectomy achieved significant luminal gain by removing superficial calcium without inducing major vessel injury [13]. In a single-center analysis of 162 patients with complex TASC C/D femoropopliteal lesions, Jetstream-assisted interventions achieved a procedural success rate of 99%, with 12-month freedom from target lesion revascularization of 92.6% and a bailout stent rate of 7.4% following adjunctive drug-coated balloon angioplasty [14]. Similarly, the prospective Phoenix Registry reported procedural success in 98% of cases, low perforation and embolization rates, and favorable mid-term outcomes even in complex lesions [15]. In addition, recent data have specifically addressed the use of rotational atherectomy in highly mobile vascular segments. Schöfthaler et al. [16] demonstrated that rotational atherectomy in the popliteal artery was associated with low bailout stenting rates and favorable clinically driven target lesion revascularization compared with an angioplasty-only approach. These findings suggest that plaque modification by rotational atherectomy may be particularly advantageous in mobile segments, where avoidance of permanent implants is desirable and biomechanical stress may compromise stent durability.

The Rotarex device, primarily designed for thrombectomy and removal of organized thrombus, has also demonstrated favorable outcomes in selected PAD populations. In patients with femoropopliteal in-stent restenosis, Rotarex combined with drug-coated balloon angioplasty achieved 12-month primary patency of 86.7% with low reintervention rates [17]. Large real-world series further confirmed high clinical success and significant improvement in ankle–brachial index following Rotarex-assisted recanalization in complex occlusions [18,19].

**Fig. 2.**
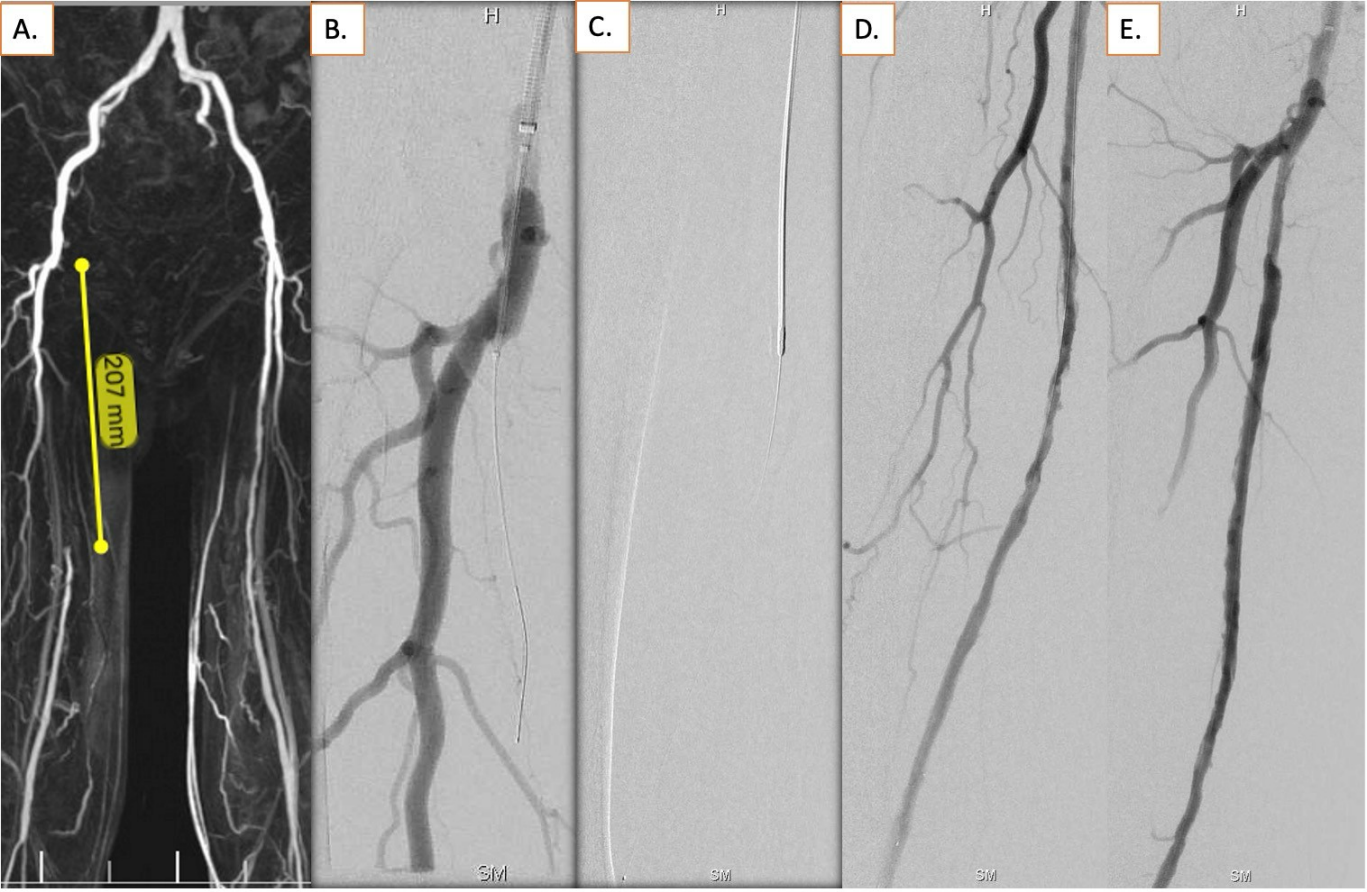
**Rotational atherectomy of an occluded SFA**. Rotational atherectomy using a 2.4 mm Jetstream catheter in a 56-year-old patient with intermittent claudication and a long (20 cm) occlusion of the superficial femoral artery (A). After antegrade recanalization (B), long-segment atherectomy was performed (C), resulting in satisfactory plaque and thrombus removal (D). Subsequent drug-coated balloon angioplasty achieved complete resolution of the stenosis with good flow and without the need for stent implantation (E).

**Fig. 3.**
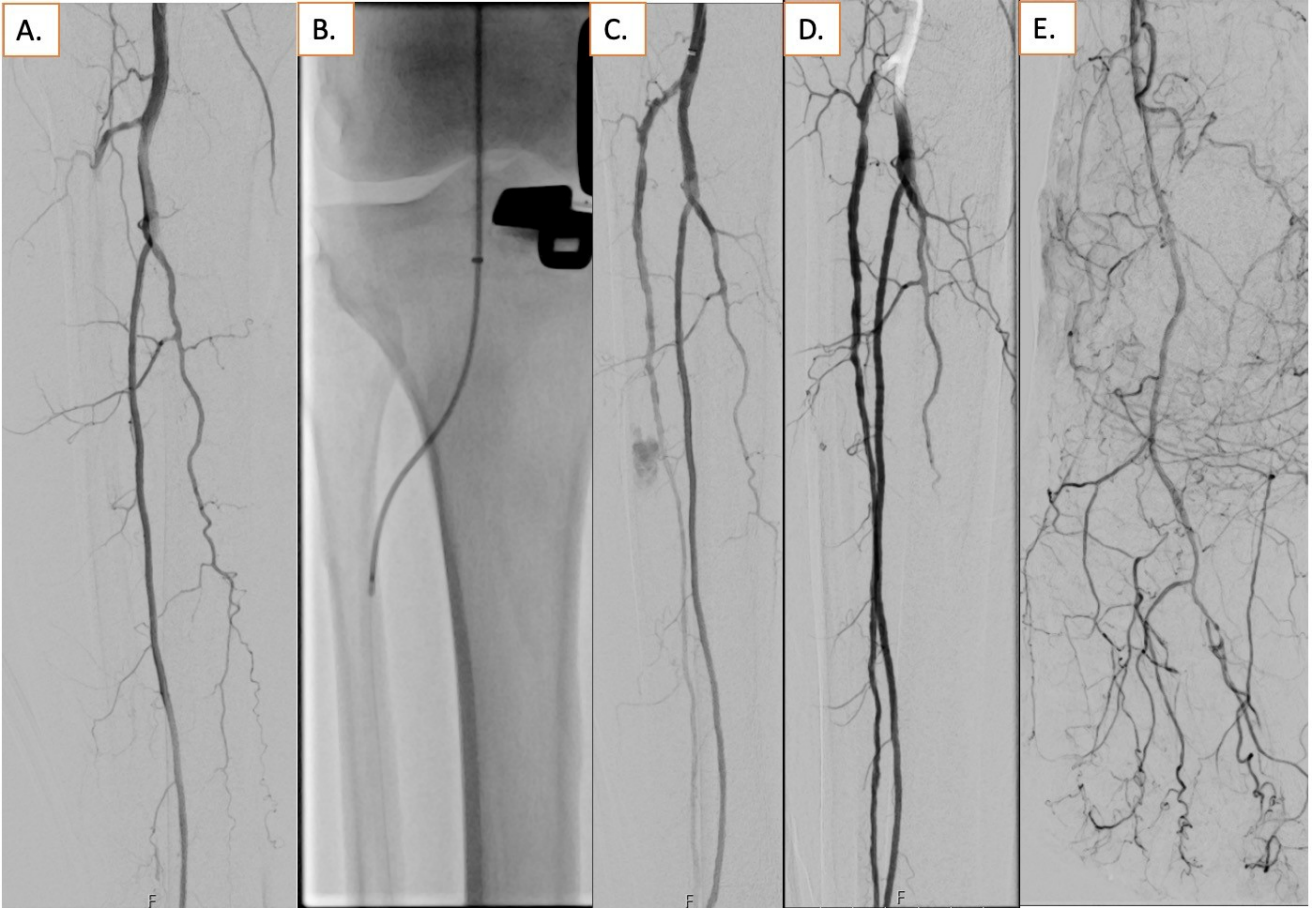
**Rotational atherectomy in BTK vessels**. Rotational atherectomy using a 1.8 mm Phoenix catheter in a 75-year-old patient (Rutherford category 5) with critical limb ischemia and an interdigital ulcer between the first and second toes. A long-segment occlusion of the anterior tibial artery with severe calcification and preserved dorsalis pedis artery is shown (A). After successful guidewire crossing, atherectomy was performed (B). The result was satisfactory; however, a paravasate occurred (C) and was managed by prolonged balloon inflation. The final angiogram (D,E) demonstrated excellent perfusion without residual stenosis and good flow to the foot.

Taken together, rotational atherectomy represents the most widely used atherectomy modality and encompasses several devices employing different mechanisms for plaque removal or pulverization. Representative clinical applications are illustrated in Figs. 2,3.

### 3.3 Laser Atherectomy

Laser atherectomy utilizes ultraviolet energy delivered through fiberoptic catheters to ablate atherosclerotic and thrombotic material. The most established system is the Turbo-Elite excimer laser catheter (Philips, Amsterdam, The Netherlands), which emits pulsed 308-nm energy and is approved for both coronary and peripheral interventions. Additional systems include the DABRA catheter (RA Medical, Carlsbad, CA, USA) and the Auryon Atherectomy System (AngioDynamics, Latham, NY, USA), which employs a 355-nm solid-state laser capable of ablating plaque of varying composition, including heavily calcified tissue.

Clinical data indicate favorable procedural and mid-term outcomes, particularly in combination with drug-coated balloon angioplasty. In patients with long and complex femoropopliteal occlusions, excimer laser atherectomy followed by DCB angioplasty achieved high technical success and encouraging 12-month patency rates [20]. More recently, first-in-human evaluation of a 355-nm solid-state laser system demonstrated high procedural success, absence of device-related major adverse events, and promising short-term patency [21].

### 3.4 Orbital Atherectomy

Orbital atherectomy is based on a diamond-coated eccentric crown that rotates at high speed, generating an elliptical orbit to progressively enlarge the vessel lumen while producing micron-sized particles. In peripheral interventions, the Stealth 360° Orbital Atherectomy System (Abbott, Abbott Park, IL, USA) is the most widely used platform, applied in both femoropopliteal and infrapopliteal disease. The FreedomFlow system has recently been introduced, aiming to improve procedural efficiency and cost-effectiveness. Orbital atherectomy is primarily used for the treatment of severely calcified lesions.

Randomized evidence is available from the COMPLIANCE 360°, CALCIUM 360, and OPTIMIZE BTK trials. Collectively, these studies demonstrated that orbital atherectomy improves vessel compliance, facilitates low-pressure balloon angioplasty, and reduces dissection rates and bailout stenting. These consistent procedural advantages support its role as an effective vessel preparation tool in heavily calcified disease [22,23,24].

However, intravascular ultrasound studies have suggested a limited capacity for lumen enlargement compared with directional techniques, which may contribute to higher bailout stenting rates in certain lesion subsets [25]. These observations highlight the importance of appropriate patient and lesion selection when applying orbital atherectomy in clinical practice.

## 4. Evidence for Atherectomy

The evidence base supporting atherectomy in peripheral artery disease is heterogeneous and remains challenging to interpret. This is partly attributable to the wide spectrum of available devices and techniques, each characterized by distinct mechanisms of action and intended clinical applications. In addition, only a limited number of randomized controlled trials have been conducted, while the majority of published data derive from registries and retrospective analyses. Clinical outcomes are further influenced by lesion morphology—ranging from thrombotic or fibrotic stenoses to heavily calcified lesions and chronic occlusions—as well as by operator experience. Despite these limitations, multiple studies suggest that atherectomy may offer procedural advantages in selected patient and lesion subsets.

### 4.1 Randomized Controlled Trials (RCTs) for Atherectomy

The randomized evidence for atherectomy is limited and has consistently shown that, although atherectomy is feasible and generally safe, it has not demonstrated superiority over balloon angioplasty, stenting, or drug-coated balloon angioplasty with respect to long-term patency or limb-related outcomes. Meta-analyses of randomized trials have confirmed these neutral long-term results while consistently identifying procedural advantages, including higher technical success and lower bailout stenting rates.

Early randomized trials compared directional atherectomy with plain balloon angioplasty in femoropopliteal disease. In the study by Vroegindeweij et al. [26], balloon angioplasty resulted in higher one-year patency than Simpson directional atherectomy, although both strategies achieved comparable clinical improvement. Similar findings were reported by Tielbeek et al. [27], who observed no significant differences in angiographic or clinical outcomes at two years, again with a nonsignificant trend toward better patency after balloon angioplasty. These early studies established the technical feasibility of atherectomy but failed to demonstrate durable superiority.

Subsequent trials in the modern device era focused increasingly on lesion preparation rather than definitive therapy. Shammas et al. [28] compared SilverHawk directional atherectomy plus adjunctive balloon angioplasty with balloon angioplasty alone and reported similar one-year target lesion revascularization rates, but a marked reduction in bailout stenting in the atherectomy group. The CALCIUM 360 trial extended this concept to heavily calcified infrapopliteal disease, demonstrating that orbital atherectomy facilitated lower balloon inflation pressures, fewer dissections, and numerically fewer bailout stents, without improving long-term patency [23].

Comparable findings were observed in the COMPLIANCE 360° trial, which randomized patients with calcified femoropopliteal lesions to orbital atherectomy plus balloon angioplasty versus balloon angioplasty alone. While the composite endpoint of adjunctive stenting or revascularization was significantly reduced at six months in the atherectomy group, primary patency and clinical improvement were similar at twelve months, indicating that early procedural benefits did not translate into sustained long-term advantage [22].

The ISAR-STATH trial broadened the comparative landscape by including drug-eluting balloon angioplasty and stenting. In this study, paclitaxel-coated balloon angioplasty combined with stenting outperformed both balloon angioplasty and atherectomy-based strategies in terms of restenosis, while atherectomy did not confer an advantage over balloon angioplasty alone [29].

More recent trials have evaluated atherectomy as an adjunct to drug delivery. In the DEFINITIVE AR study, directional atherectomy combined with a drug-coated balloon significantly improved acute procedural outcomes, including fewer dissections and reduced bailout stenting, but did not improve one-year patency or reintervention rates compared with drug-coated balloon angioplasty alone [30]. Similar results were reported by Cai et al. [31] in patients with long femoropopliteal lesions. The OPTIMIZE BTK trial further supported the role of orbital atherectomy as a vessel preparation tool in heavily calcified infrapopliteal disease, demonstrating high procedural success and favorable mid-term outcomes without statistically significant differences in long-term patency [24].

Randomized data on rotational atherectomy remain sparse. The JET-RANGER trial, the only randomized study in this field, was terminated prematurely and therefore underpowered. Nevertheless, it demonstrated clear procedural advantages of rotational atherectomy combined with drug-coated balloon angioplasty, particularly with respect to procedural success and reduced bailout stenting [32].

Taken together, randomized trials consistently demonstrate that atherectomy—whether directional, orbital, or rotational—does not confer superior long-term outcomes compared with contemporary endovascular strategies. However, across multiple studies and device platforms, atherectomy reproducibly improves acute procedural results, including greater luminal gain, lower dissection rates due to reduced barotrauma, and diminished need for bailout stenting. These effects may be particularly relevant in mobile vascular segments and anatomically challenging zones, where avoidance of permanent implants is desirable.

### 4.2 Non-Randomized Studies for Atherectomy

Beyond the limited randomized evidence, a substantial body of non-randomized, predominantly observational data has reported favorable procedural and clinical outcomes associated with atherectomy. These studies, recently summarized by Carr et al. [33], frequently included lesion subsets that were largely underrepresented or excluded from randomized trials, such as heavily calcified disease, long femoropopliteal occlusions, and in-stent restenosis.

In calcified lesions, the procedural advantages observed in randomized trials—particularly lower dissection rates and reduced bailout stenting—have been consistently confirmed in real-world studies. The prospective DEFINITIVE Ca++ trial demonstrated the safety and feasibility of directional atherectomy using the SilverHawk/TurboHawk system with distal embolic protection in severely calcified femoropopliteal arteries, achieving high acute success and meaningful symptomatic improvement [11]. These findings were further corroborated by the VIVA REALITY study, which reported low provisional stenting rates and favorable 12-month outcomes following directional atherectomy combined with drug-coated balloon angioplasty in a cohort characterized by long lesion length, a high prevalence of chronic total occlusions (CTOs), and extensive bilateral calcification [34].

For long and complex femoropopliteal occlusions, rotational atherectomy has emerged as a particularly relevant strategy. Recent analyses by Dukic et al. [14] and Taneva et al. [35] demonstrated that Jetstream-assisted interventions combined with drug-coated balloon angioplasty achieved high technical success, low bailout stent rates, and encouraging mid-term patency, even in predominantly TASC C/D lesions., These observations support the role of rotational atherectomy as an effective vessel preparation tool in extensive occlusive disease.

In the setting of in-stent restenosis, and especially total in-stent occlusions, outcomes with balloon angioplasty alone are known to be suboptimal, as highlighted by the PACUBA trial [36]. In this context, atherectomy appears to represent a valuable adjunctive strategy. Several studies have shown that combining rotational atherectomy with drug-coated balloon angioplasty yields acceptable patency and reintervention rates even in these challenging scenarios [37,38]. However, these data also underscore the importance of distal embolic protection, particularly when treating ISR with high plaque burden.

Mechanistic insights further support the clinical observations. Preclinical work by Tzafriri et al. [39] demonstrated that plaque modification by atherectomy enhances local drug uptake and facilitates deeper penetration of paclitaxel into the vessel wall, providing a plausible biological rationale for combining atherectomy with antiproliferative therapies.

Taken together, non-randomized data consistently suggest that atherectomy offers meaningful procedural and clinical advantages in complex lesion subsets that are poorly represented in randomized trials. While these findings are inherently limited by selection bias and confounding, they help explain the continued use of atherectomy in daily practice and support its role as a complementary vessel preparation strategy in carefully selected patients with advanced peripheral artery disease.

### 4.3 Current Guidelines

Given the heterogeneous and partly conflicting evidence base, current guideline recommendations regarding atherectomy vary across professional societies. The 2024 European Society for Vascular Surgery (ESVS) guidelines on peripheral arterial disease adopt a cautious stance and assign a Class III recommendation against the routine use of atherectomy [40]. This position is largely based on randomized trials and earlier-generation technologies, many of which were conducted in selected populations and small single-center settings. Importantly, the guideline wording allows for selective use in individual cases, particularly when lesion morphology may justify plaque modification.

In contrast, the updated 2024 German S3 (AWMF) guideline adopts a more permissive and pragmatic approach. Under the broader concept of “vessel preparation”, atherectomy is explicitly acknowledged as a potential adjunctive strategy in both native vessels and in-stent restenosis, provided it is applied in a targeted manner by experienced operators and in appropriately selected lesions [41].

The 2024 American Heart Association/American College of Cardiology (AHA/ACC) guidelines for the management of lower extremity peripheral artery disease provide comprehensive recommendations on endovascular revascularization but do not issue a specific statement on atherectomy [42]. Instead, they refer to complementary documents, including the Society for Vascular Surgery (SVS) Appropriate Use Criteria, which do not constitute formal guideline recommendations but offer scenario-based appropriateness ratings [43]. Within this framework, atherectomy is classified as “may be appropriate” in selected clinical contexts when anticipated benefits outweigh procedural risks.

Similarly, the Society for Cardiovascular Angiography and Interventions (SCAI) consensus document recognizes atherectomy as an adjunctive vessel preparation tool in selected moderate to severely calcified lesions and specific anatomical scenarios, including the ostial SFA and the popliteal artery, while explicitly discouraging its use as a stand-alone definitive treatment strategy [44].

Taken together, these differing recommendations reflect the ongoing uncertainty and heterogeneity of the evidence base. While several guidelines discourage routine use, others acknowledge a selective role for atherectomy as part of lesion preparation in complex anatomy. This divergence underscores both the procedural potential of atherectomy and the continued need for high-quality data to better define its appropriate clinical application.

## 5. Critical Appraisal

Despite its widespread use, atherectomy has been subject to substantial criticism. Early concerns were raised by Chung et al. [45], who reported poor mid-term patency following directional atherectomy with the SilverHawk device in infrainguinal disease, with most patients developing restenosis or occlusion within 12 months. Although these data reflect earlier-generation devices and historical practice patterns, they highlighted fundamental issues regarding durability and the potential need for repeated interventions.

Randomized evidence has further contributed to this critical perspective. Two randomized controlled trials evaluating directional and orbital atherectomy in infrapopliteal arteries failed to demonstrate incremental benefit with respect to patency, reintervention, or amputation rates [24,46]. As a consequence, many operators remain cautious regarding the use of atherectomy below the knee, particularly given the small vessel diameter and the limited margin for procedural complications.

More contemporary observational data have also questioned the role of atherectomy in femoropopliteal disease. An analysis from the Vascular Quality Initiative by Bai et al. [47], encompassing more than 38,000 femoropopliteal interventions, found no improvement in clinical outcomes with atherectomy compared with balloon angioplasty or stenting. Although atherectomy was associated with lower dissection rates and, in some comparisons, higher one-year patency than balloon angioplasty alone, it was also linked to higher rates of distal embolization, more frequent reinterventions, and—in certain analyses—higher amputation rates. Importantly, these findings are derived from retrospective, non-randomized data and are therefore subject to significant confounding and selection bias.

These observations are consistent with a recent systematic review and network meta-analysis by Yiu et al. [48], which found no significant advantage of atherectomy over balloon angioplasty or intravascular lithotripsy when used as vessel preparation prior to drug-coated balloon angioplasty. Across studies, the only consistently observed benefit was a reduction in bailout stenting, a finding that has also been uniformly reported in randomized trials and meta-analyses [49].

More recent comparative evidence further challenges the incremental benefit of atherectomy-based vessel preparation strategies. In a contemporary meta-analysis of complex femoropopliteal disease, Cosacco et al. [50] demonstrated that a primary drug-coated balloon strategy—frequently requiring adjunctive procedures such as atherectomy and bailout stenting—did not result in superior clinical outcomes compared with primary implantation of contemporary drug-eluting stents. These findings suggest that, even when extensive vessel preparation is applied, atherectomy does not necessarily translate into improved mid-term patency compared with optimized stent-based therapy [50]. However, long-term data on the consequences of long permanent metallic implants in the femoropopliteal segment are still warranted.

Several unresolved technical considerations further limit broad application. In complex occlusions requiring subintimal recanalization, it remains unclear whether effective plaque debulking can be achieved, as device interaction with true luminal atherosclerotic tissue may be limited. In this direction, a panel of experts from different countries and disciplines recently warned against its use in long CTOs, which cannot be passed intraluminally [51]. In addition, alternative treatment strategies must be acknowledged: biomimetic interwoven nitinol stents, most notably the Supera stent, have demonstrated favorable outcomes in off-label and real-world studies of mobile segments such as the popliteal artery, representing a valid alternative in selected cases. However, comparative data on the use of a primary interwoven stent versus a debulking, stent-avoiding strategy combined with DCB for the popliteal artery are scarce.

Beyond clinical outcomes, atherectomy has also been scrutinized from a health-policy and economic perspective. In the United States, utilization increased markedly over recent years, particularly in ambulatory surgical centers. Analyses of Medicare data revealed substantial variation in practice patterns, with some providers performing atherectomy at disproportionately high rates, including in less complex lesions [52,53]. These findings have raised concerns that reimbursement structures may have influenced utilization beyond what is supported by the available evidence.

In summary, while atherectomy reproducibly reduces dissections and the need for bailout stenting, robust evidence for durable long-term benefit remains lacking. Procedural risks, including distal embolization, must be carefully weighed, and economic considerations cannot be ignored [54]. These limitations underscore the importance of restrained, evidence-informed, and highly selective use of atherectomy in contemporary endovascular practice.

At the same time, randomized trials and real-world data have reproducibly demonstrated procedural advantages associated with atherectomy. Across studies, atherectomy has been shown to improve acute luminal gain, reduce balloon inflation pressures, lower dissection rates, and decrease the need for bailout stenting. These effects reflect reduced barotrauma and effective plaque modification and are particularly relevant in heavily calcified lesions, long-segment occlusions, and anatomically challenging or highly mobile vascular segments where permanent implants may be undesirable.

Non-randomized studies and large registries further suggest that these procedural advantages may translate into clinically meaningful benefits in selected, complex lesion subsets that are underrepresented in randomized trials, including severe calcification and in-stent restenosis. However, these observations must be interpreted with caution, given the inherent limitations of observational data, including selection bias, confounding, and center-specific practice patterns.

The role of atherectomy must also be considered in the context of alternative vessel preparation strategies. Intravascular lithotripsy, drug-coated balloon angioplasty, and contemporary stent designs have expanded the therapeutic armamentarium and, in selected scenarios, offer excellent outcomes with favorable safety profiles. Rather than competing technologies, these approaches should be viewed as complementary, with atherectomy representing one option within a broader, increasingly individualized endovascular strategy.

## 6. Conclusion

**Fig. 4.**
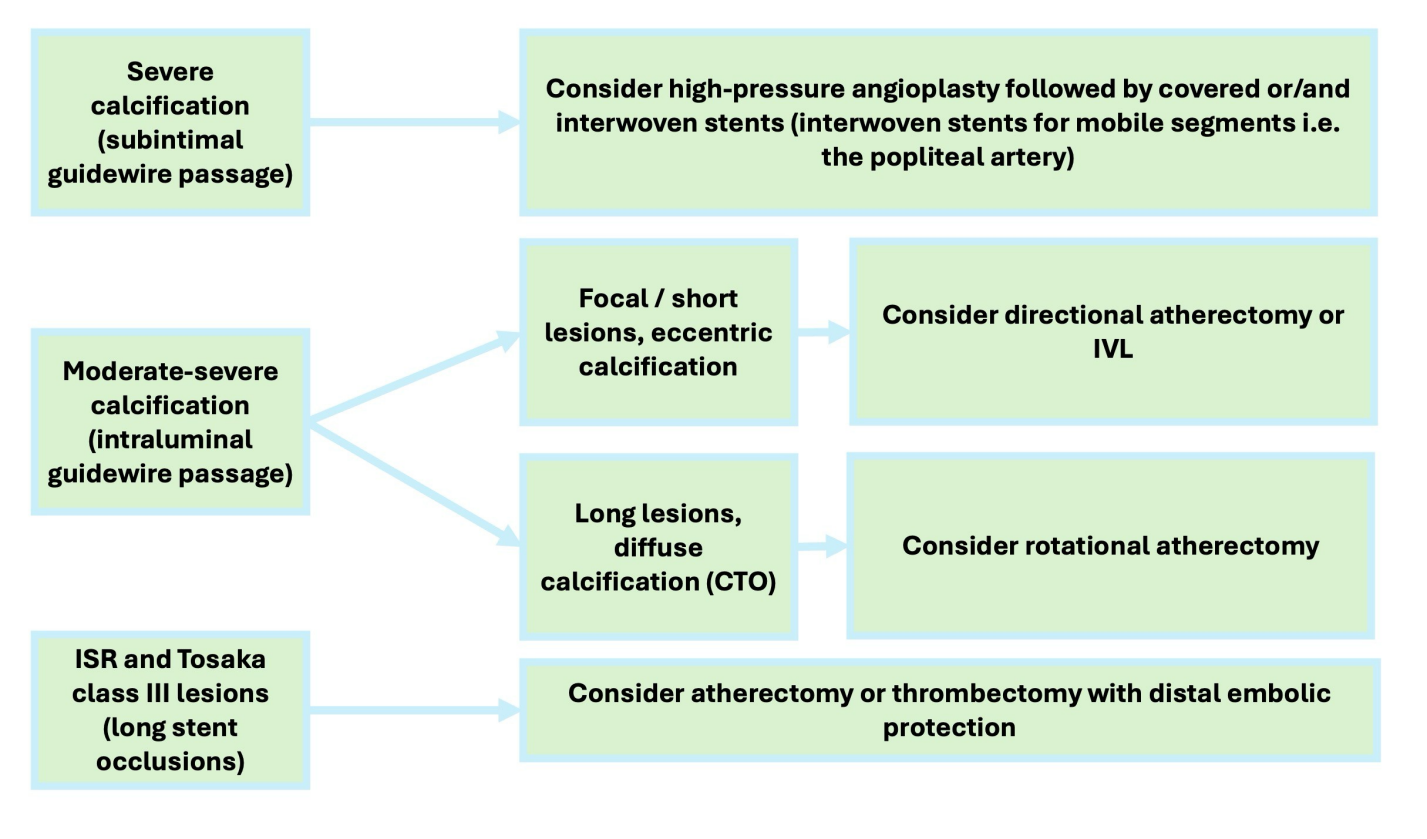
**Patient-centered and anatomy-driven use of atherectomy in selected segments and lesion morphologies**.

The available evidence on atherectomy in peripheral artery disease remains heterogeneous and, in part, conflicting. Randomized controlled trials across different device platforms and anatomical segments have consistently confirmed its ability for lumen gain without barotrauma, resulting in lower dissection rates and lower rates of bail-out stenting, but also failed to demonstrate its superiority over balloon angioplasty, drug-coated balloons, or contemporary stent-based strategies with respect to long-term patency, freedom from reintervention, or limb-related outcomes. Procedure-specific risks, including distal embolization and health-economic considerations, do not support the routine use of atherectomy but rather its anatomy-driven use in selected segments and lesion morphologies and patients with adequate distal run-off (Fig. 4). Its responsible use requires careful integration of available evidence, operator expertise, and consideration of alternative technologies. Future research focusing on morphology-guided patient selection and imaging-supported strategies is essential to better define its optimal role in contemporary endovascular practice.
